# Global analysis of a time fractional order spatio-temporal SIR model

**DOI:** 10.1038/s41598-022-08992-6

**Published:** 2022-04-06

**Authors:** Moulay Rchid Sidi Ammi, Mostafa Tahiri, Mouhcine Tilioua, Anwar Zeb, Ilyas Khan, Mulugeta Andualem

**Affiliations:** 1 Department of Mathematics, AMNEA Group, FST Errachidia, Moulay Ismail University of Meknes, P.O. Box 509, Boutalamine, 52000 Errachidia Morocco; 2 Department of Mathematics, COMSATS University Islamabad, Abbottabad, 22060 Khyber Pakhtunkhwa Pakistan; 3 Department of Mathematics, College of Science Al-Zulfi, Majmaah University, Al-Majmaah, 11952 Saudi Arabia; 4 MAIS Lab., MAMCS Group, FST Errachidia, Moulay Ismail University of Meknes, P.O. Box 509, Boutalamine, 52000 Errachidia, Morocco; 5 Department of Mathematics, Bonga University, Bonga, Ethiopia

**Keywords:** Computational biology and bioinformatics, Diseases

## Abstract

We deal in this paper with a diffusive *SIR* epidemic model described by reaction–diffusion equations involving a fractional derivative. The existence and uniqueness of the solution are shown, next to the boundedness of the solution. Further, it has been shown that the global behavior of the solution is governed by the value of $R_0$, which is known in epidemiology by the basic reproduction number. Indeed, using the Lyapunov direct method it has been proved that the disease will extinct for $R_0 <1$ for any value of the diffusion constants. For $R_0>1$, the disease will persist and the unique positive equilibrium is globally stable. Some numerical illustrations have been used to confirm our theoretical results.

## Introduction

In many sciences, experiments can be done to collect information and test hypotheses. Doing experiments for testing the outbreak of infection in different populations is generally not possible, immoral, and costly^1^. In most cases, the data are often unprecise due to underreporting. This deficit in the data collections makes a reliable estimation of the parameters impossible, which is noted hugely in our recent fight against the pandemic of COVID-19 disease. Then, it is only possible to approximate certain parameters. Based on the fact that repeatable experiments are not available in epidemiology, mathematical modeling and numerical simulations can be used to perform the theoretical experiments needed for a variety of parameter values^2^. They also help to understand, analyzing and limiting the outbreak of infectious diseases^3^. So we should be able to give a response to important issues as:

- The possibility of having an epidemic.
- The knowledge of the duration of this epidemic is important, for determining the proper public health intervention.
- The density of the individuals that have been touched by this disease.
- The type of control that allows authorities to make decisions about strategies as isolation, quarantine, vaccination, and treatment.

In this context, most *SIR* models have been traditionally investigated in an uniform distribution of populations which are generally formulated only by ordinary differential equations. This fact shows the possibility that the disease can outbreak over a spatial region. In reality the infected individuals have the greatest effect on spatially nearest susceptible persons. The outbreak of infectious diseases is influenced by the spatial movement of populations. The great development in transportation networks is among the main contributing factors in the growth of people’s movement around the world. For these reasons, many recent researches have been devoted to the study of reaction–diffusion models (particularly the existence, uniqueness, positivity and stability of the equilibria). They have as goal predicting the evolution of diseases in relation to time and space simultaneously^4^.

Recently, fractional derivatives have several applications in many fields such as mechanics^5^, control theory^6^, bioengineering^7^ and viscoelasticity^8^. We point out that derivative order (fractional) can be any positive real choice in order to best correspond to the available data^9^. Consequently, the systems of non-integer order differential equations or partial differential equations give a more realistic behavior^10^. Fractional-order-derivatives are used widely in epidemiology to describe disease evolution and, in most cases, are considered to be more precise than the classical derivative^11^. For example, the spread of the virus is generally discontinuous, so that they are not well described by systems of ordinary differential equations. Then fractional systems naturally deal with such a property of discontinuity^12^. In addition, different models have used fractional derivatives to better predict the outbreak of diseases with sufficient data, among these models we find *SIR* epidemic model with fractional derivative with Mittag–Leffler kernel^13^, hybrid variable-order fractional coronavirus (2019-nCov)^14^, a hybrid stochastic fractional order Coronavirus (2019-nCov)^15^. In^16^ a survey for novel fractional biological models and the numerical methods used to study these models. In^11^ the authors used the real data from the Florida Department of Health in the period from September 2011 to July 2014, they concluded that the absolute error between the solutions obtained statistically and that of the fractional model decreases more than those obtained by the model of integer derivative.

In the literature, several definitions of fractional derivatives have been used in different works^17^. Among the most popular non-integer derivatives is that of Riemann–Liouville. It is not often adequate for modeling physical systems because it does not keep the nullity of the derivative constant and the initial conditions of the Cauchy problem are given by fractional derivatives. Caputo presents another alternative preserving the derivative of the constant is null and the initial conditions remain expressed as in the classical case by derivatives of integer order^18^.

The novelties of this work can be summarized in the following two points: Firstly, we investigate the global behavior of more real extension’s of a basic *SIR* model with memory effects measured by Caputo’s fractional derivative in time of order $0 <\alpha \le 1$, such that when $\alpha = 1$ we obtain a classical model (without memory). More precisely, memory measured by the nonlocal operator of the fractional derivative highlights the other possibilities not included in the model formulation as fear from infection and the movement in the space, and closing stores, reducing the mobility of persons, and others, which makes fractional systems more realistic to describe the real life situations. Secondly, we have constructed Lyapunov functions to show the global stability of the equilibrium points in a more general framework where the proposed system takes into account the spatial behavior of populations and memory effect. Through this research, we show that our system is well posed in the sense that we prove the global existence, uniqueness and boundedness of the solution. By constructing suitable Lyapunov functions, the disease will be eradicated for $R_0\le 1$, and persist for $R_0>1$.

This research is structured as follows. In “Preliminaries” section, we remember some basic results for fractional calculus. The proposed model formulation is given in “Model formulation” section. Next, we show in “Existence and uniqueness of solutions” section the existence and uniqueness of a bounded solution. “Existence of equilibria and local stability” section is devoted for calculating all possible equilibrium states. The global behavior of the solution is the subject of interest in “Global stability” section. Numerical simulations of the considered model in agreement with theoretical results are illustrated in “Graphical representation” section.

## Preliminaries

We now recall definitions of the Mittag–Leffler function and Caputo fractional time derivative. First, the Mittag–Leffler function, $E_{\alpha }(z)$, is defined as the family of entire functions of *z* given as

$$\begin{aligned} E_{\alpha }(z) = \sum _{k=0}^{\infty } \frac{z^{k}}{\Gamma (k\alpha + 1)}, \quad {\alpha >0}, \quad z \in \mathbb {C}, \end{aligned}$$

whenever the series converges^19^, where $\Gamma (\cdot )$ is Gamma function .

Observe that the Mittag–Leffler function generalizes the exponential function: $E_{1}(z)=exp(z)$.

### Definition 2.1

(^17^) We consider $f\in L^1(\mathbb {R}^+)$. The Riemann–Liouville fractional-integral of order $\alpha > 0$ of *f* is

$$\begin{aligned} I^\alpha f(t) = \int _0^t M(t,s) f(s) ds, \end{aligned}$$

where $M(t,s)=\dfrac{1}{\Gamma (\alpha )}(t-s)^{\alpha -1}$ is a power law function.

### Definition 2.2

(^17^) We consider $\alpha > 0$, and letting $n \in \mathbb {N}$ such that $n - 1 < \alpha \le n$. The fractional derivative in sense of Caputo for $\alpha$ for a function $f \in C^{n}([0, +\infty ), \mathbb {R})$ is

$$\begin{aligned} ^{C}_{0}D{_t^{\alpha }} f(t) = I^{n-\alpha } D^{n} f(t) = \dfrac{1}{\Gamma (n-\alpha )} \int _0^t \dfrac{f^{(n)} (s)}{(t-s)^{\alpha +1-n}} ds, \end{aligned}$$

with $D = \dfrac{d}{dt}$ for $n=1$. In particular, for $0< \alpha < 1$, we get

$$\begin{aligned} ^{C}_{0}D{_t^{\alpha }} f(t) = \dfrac{1}{\Gamma (1-\alpha )} \int _0^t \dfrac{f' (s)}{(t-s)^{\alpha }} ds. \end{aligned}$$

For more details about the definition of fractional derivative in sense Caputo , we refer to^17^.

## Model formulation

Let $\Omega$ a bounded set in ${\mathbb {R}}$ with smooth boundary $\partial \Omega$, and [0, *T*] is a finite interval. The classic *SIR* epidemic model^20^ governed by reaction–diffusion equations takes the following form: for ${(t,x)}\in Q_{T}=[0, T]\times \Omega$

3.1 $$\begin{aligned} \begin{aligned} \frac{\partial S{(t,x)}}{\partial t} - \lambda _1\Delta S{(t,x)}&= \Lambda -\beta S{(t,x)} I{(t,x)}-\mu S{(t,x)},\\ \frac{\partial I{(t,x)}}{\partial t} - \lambda _2\Delta I{(t,x)}&= \beta S{(t,x)} I{(t,x)}-(\mu +r)I{(t,x)},\\ \frac{\partial R{(t,x)}}{\partial t} - \lambda _3\Delta R{(t,x)}&= r I{(t,x)}- \mu R{(t,x)}{.} \end{aligned} \end{aligned}$$

The total population *N* is divided into three compartments of the pathological state where *S*(*t*, *x*), *I*(*t*, *x*) and *R*(*t*, *x*) are respectively the densities of the susceptible population, infected population, removed population at time *t* and the spatial location *x*.

The positive constants $\Lambda$, *r* and $\mu$ are respectively the entering flux into S-class, the recovery rate and the natural death rate. Persons in S-class acquire infection after a direct contact with person in I-class, with the rate $\beta SI$, where $\beta$ is the transmission coefficient per unit of time. Positive constants $\lambda _1,\lambda _2$ and $\lambda _3$ denote the diffusion coefficients for the susceptible, infected and removed individuals, respectively. $\Delta$ represents the usual Laplacian operator.

In the actual world, and during an epidemic, there are numerous components that varies and can influence the outbreak of a disease and cannot be included in the model formulation, as the fear generated by the population from infection, weather (specific change in the weather help or reduce the spread of disease). This phenomena can be modeled by replacing the ordinary differential derivative by a fractional one, as it is used in understanding many real world phenomena, we cite for instance the research^21^. For the model (3.1), the state at any time *t* does not depend on the previous history. It is a markovian process (memory depends on time and corresponds to a Dirac function $\delta (t,s)$). However, the evolution and control of epidemic processes in human societies cannot be envisaged without any memory effect. When an idea is propagated within a human population, the experience or knowledge of individuals on this idea should influence their responses. To incorporate long-term memory effects in the classical *SIR* model (3.1), we convert it to an equivalent system of integral equations. We change the function $\delta (t,s)$ by the power law function *M*(*t*, *s*) which shows a slow decay so that the state of the system at early times also contributes to the evolution, afterward applying the fractional Caputo derivative^22^. One of our goals is to study the effect of vaccination on the basic reproduction number. We therefore introduced the term vaccination *u* into model (3.1). It is assumed that vaccination converts susceptible individuals into the removed class and confers immunity on them. Motivated by the above discussion, we introduce the time fractional derivative to the diffusive *SIR* model in the following manner:

3.2 $$\begin{aligned} \begin{aligned} ^{C}_{0}D{_t^{\alpha }}S{(t,x)}&= \lambda _1\Delta S{(t,x)}+\Lambda -\beta S{(t,x)} I{(t,x)}-\mu S{(t,x)}-uS{(t,x)},\\ ^{C}_{0}D{_t^{\alpha }}I{(t,x)}&= \lambda _2\Delta I{(t,x)} +\beta S{(t,x)} I{(t,x)}-(\mu +r)I{(t,x)}, \, {(t,x)}\in Q_{T},\\ ^{C}_{0}D{_t^{\alpha }}R{(t,x)}&= \lambda _3\Delta R{(t,x)} + r I{(t,x)}- \mu R{(t,x)} + uS{(t,x)}. \end{aligned} \end{aligned}$$

We consider that the model (3.2) is self-contained and there is a dynamic across the boundary but there is no emigration. Then the no-flux homogeneous Neumann boundary conditions are

3.3 $$\begin{aligned} \frac{\partial S{(t,x)}}{\partial \nu }=\frac{\partial I{(t,x)}}{\partial \nu }=\frac{\partial R{(t,x)}}{\partial \nu }=0, \quad {(t,x)}\in \Sigma _{T}=[0, T]\times \partial \Omega . \end{aligned}$$

For epidemiological aspect, we consider that the following initial conditions of the three classes are positive

3.4 $$\begin{aligned} S(0,x)=S_0, I(0,x)=I_0 \, and \, R(0,x)=R_0, \, x \in \Omega . \end{aligned}$$

The constant *u* refers to the vaccination rate. It is presumed that the vaccination transforms the persons in S-class to the removed class after acquiring immunity. We denote by $\nu$ the outward unit normal vector on the boundary $\partial \Omega$ and by $\frac{\partial }{\partial \nu }=\nu . \nabla$ the normal derivative.

## Existence and uniqueness of solutions

Letting $\mathbb {X}=C(\bar{\Omega },\mathbb {R})$ and $X^3$ be Banach spaces endowed by the uniform norms

$$\begin{aligned} \Vert h\Vert _{\mathbb {X}}=\underset{x \in \Omega }{sup} |h(x)|, \, \forall h\in \mathbb {X}, \end{aligned}$$

and

$$\begin{aligned} \Vert H\Vert _{\mathbb {X}^3}=\underset{x \in \Omega }{sup} \Vert H(x)\Vert _1, \, \forall H\in \mathbb {X}^3, \end{aligned}$$

with $\Vert H(x)\Vert _1 = \sum _{i=1}^{3}|H_i(x)|$ is the Manhattan norm^23^.

We set $J=(J_{1}, J_{2}, J_{3})$, $J^{0}=(J_{1}^{0}, J_{2}^{0}, J_{3}^{0})$, $\lambda =(\lambda _{1}, \lambda _{2}, \lambda _{3})$ and we assume that *A* is the linear diffusion operator where

$$\begin{aligned}&A: D(A) \subset \mathbb {X}^{3} \rightarrow \mathbb {X}^{3} \\&AJ = \lambda \Delta J = ( \lambda _{1} \Delta J_{1}, \lambda _{2} \Delta J_{2}, \lambda _{3} \Delta J_{3}), \quad \forall J \in D(A), \end{aligned}$$

where

$$\begin{aligned} D(A)= \left\{ J \in \mathbb {X}^{3}: \, \Delta J\in \mathbb {X}^{3}, \, \frac{\partial J}{\partial \nu }=0_{{\mathbb {R}}^3} \, \text{ for } x \in \partial \Omega \right\} . \end{aligned}$$

Let the function *f* defined by $f : [0, T]\times \mathbb {X}^{3}\mapsto \mathbb {X}^{3}$, where

$$\begin{aligned} f(t,J(t)):=f(J(t))= ( f_{1}(J(t)), f_{2}(J(t)), f_{3}(J(t))), \end{aligned}$$

with

$$\begin{aligned} {\left\{ \begin{array}{ll} f_{1}(J(t))= &{} \Lambda - \beta J_{1}J_{2}-\mu J_{1}-u J_{1},\\ f_{2}(J(t))= &{} \beta J_{1} J_{2} -(\mu +r) J_{2}, \qquad t \in [0, T], \\ f_{3}(J(t)) = &{} r J_{2}- \mu J_{3} +u J_{1}. \end{array}\right. } \end{aligned}$$

The model can expressed as

4.1 $$\begin{aligned} {\left\{ \begin{array}{ll} ^{C}_{0}D_{t}^{\alpha }J = &{} A J + f(J(t)),\\ J(0)= &{}J^{0}, \end{array}\right. } \end{aligned}$$

where $$J=(S,I,R)$$ and $$J^0=(S_0,I_0,R_0)$$.

### Proposition 4.1

Let $0<\alpha \le 1$, for any $J^0 \in D(A)$, problem (4.1) has a unique non-negative solution $J\in C([0, T];{X}^{3})$ given by

$$\begin{aligned} J(t)= \int _{0}^{\infty }\Phi _{\alpha }(\theta )Q(t^{\alpha }\theta )J^0d\theta + F(t), \end{aligned}$$

where

4.2 $$\begin{aligned} F(t)=\alpha \int _{0}^{t}\int _{0}^{\infty }\theta (t-\tau )^{\alpha -1}\Phi _{\alpha }(\theta )Q((t-\tau )^{\alpha }\theta )f(\tau )d\theta d\tau \end{aligned}$$

and $\Phi _{\alpha }(\theta )$ is a probability density function defined on $(0,\infty )$.

### Proof

Since *A* is a linear closed operator defined on a dense set *D*(*A*) in $\mathbb {X}^{3}$ into itself, then it generates a $C_0$-semigroup $\{Q(t), t\ge 0\}$ of contractions on $\mathbb {X}^{3}$. It is known that function *f* is Lipshitz continuous in *y* uniformly with respect to $t\in [0, T]$ if $y_i\ge 0$ for $i=1, 2$ and 3. Using^24^, Theorem 3.1], we have the existence and uniqueness results. $\square$

It remains to show that the solution is bounded. By summing the three equations of system (3.2), we get

$$\begin{aligned} ^{C}_{0}D{_t^{\alpha }}S{(t,x)}+\,^{C}_{0}D{_t^{\alpha }}I{(t,x)}+\,^{C}_{0}D{_t^{\alpha }}R{(t,x)}= \lambda _1\Delta S{(t,x)}+\lambda _2\Delta I{(t,x)}+ \lambda _3\Delta R{(t,x)}\\ \quad +\Lambda -{\mu }(S{(t,x)}+I{(t,x)}+R{(t,x)}). \end{aligned}$$

Integrating in $\Omega$ the two sides of the above equality, we have

$$\begin{aligned} \int _{\Omega }\,\bigg (\, ^{C}_{0}D{_t^{\alpha }}S{(t,x)}+\,^{C}_{0}D{_t^{\alpha }}I{(t,x)}+\,^{C}_{0}D{_t^{\alpha }}R{(t,x)}\bigg ) dx= \int _{\Omega }\,\bigg (\,\lambda _1\Delta S{(t,x)}+\lambda _2\Delta I{(t,x)} \\ \quad +\lambda _3\Delta R{(t,x)}\bigg ) dx +\int _{\Omega }\,\bigg (\,\Lambda -\mu (S{(t,x)}+I{(t,x)}+R{(t,x)})\bigg ) dx. \end{aligned}$$

Applying Green’s formula and using the homogeneous Neumann boundary conditions (3.3) we get

$$\begin{aligned}&\int _{\Omega }\, \bigg (\, ^{C}_{0}D{_t^{\alpha }}S{(t,x)}+\,^{C}_{0}D{_t^{\alpha }}I{(t,x)}+\,^{C}_{0}D{_t^{\alpha }}R{(t,x)}\bigg )dx\\&\quad =\int _{\Omega }\,\bigg (\Lambda -\mu (S{(t,x)}+I{(t,x)}+R{(t,x)})\bigg ) dx\\&\quad =\Lambda |\Omega |-\mu \int _{\Omega }\,\bigg (S{(t,x)}+I{(t,x)}+R{(t,x)}\bigg ) dx. \end{aligned}$$

Note that

$$\begin{aligned} \int _{\Omega }\,\bigg (S{(t,x)}+I{(t,x)}+R{(t,x)}\bigg ) dx=N(t). \end{aligned}$$

Due to the property of the linearity of the Caputo’s operator and Fubini’s Theorem, one have

$$\begin{aligned} ^{C}_{0}D{_t^{\alpha }}N(t)=\Lambda |\Omega |-\mu N(t). \end{aligned}$$

Solving this equality by using Laplace’s transform, we obtain

$$\begin{aligned} N(t)=N(0)E_{\alpha }(-\mu t^{\alpha })+\frac{\Lambda }{\mu }(1- E_{\alpha }(-\mu t^{\alpha })). \end{aligned}$$

Due to $0\le E_{\alpha }(-\mu t^{\alpha })\le 1,$ we conclude that $N(t)\le N(0)+\frac{\Lambda }{\mu }$ hence the solution is bounded.

### Remark 4.2

Owing to^25^, Theorem 3.1], then (3.2)–(3.4) has a unique solution which is non-negative and bounded.

Noting that the two first equations does not depend on the class *R*(*t*, *x*) and then are uncoupled with the last equation of the system (3.2). Hence our attention will concentrated on the analysis of the following reduced system: for ${(t,x)}\in [0,\infty )\times \Omega$.

4.3 $$\begin{aligned} \begin{aligned} ^{C}_{0}D{_t^{\alpha }}S{(t,x)}&= \lambda _1\Delta S{(t,x)}+\Lambda -\beta S{(t,x)} I{(t,x)}-\mu S{(t,x)}-uS{(t,x)},\\ ^{C}_{0}D{_t^{\alpha }}I{(t,x)}&= \lambda _2\Delta I{(t,x)} +\beta S{(t,x)} I{(t,x)}-(\mu +r)I{(t,x)}. \end{aligned} \end{aligned}$$

## Existence of equilibria and local stability

The principal goal of this section is to determine the equilibria for (4.3). A crucial idea in epidemiology is the existence of significant threshold values quantifying and measuring an outbreak spread in a population. The given value

$$\begin{aligned} R_0 = \dfrac{\beta \Lambda }{(\mu + u)(\mu + r)} \end{aligned}$$

is the basic reproduction number^26^. It is understood as the average number of newly cases of infection, generated by an person in I-class during infectious period, in a compartment entirely composed of susceptible individuals. From the definition of $R_0$, we conclude the following results.

### Theorem 5.1

(i) There is always a disease-free equilibrium denoted $E_f=(S_f, 0)$, with $S_f = \dfrac{\Lambda }{\mu +u}$.
(ii) If $R_0 > 1$, there exists a unique endemic equilibrium denoted $E^* = \left( S^*, I^* \right) ,$ where
  $$\begin{aligned} S^*=\frac{\mu +r}{\beta }\, \,and\, \, I^*=\frac{\mu +u}{\beta }(R_0-1). \end{aligned}$$

### Proof

(i) By a straightforward computation, we get $E_f$ is a steady state of (4.3) which always exists.
(ii) To get the other equilibrium, we need to solve (4.3) for $(S, I)= (S^*, I^*)$. We then obtain $S^*=\frac{\mu +r}{\beta }\, \,and\, \, I^*=\frac{\mu +u}{\beta }(R_0-1)$. Hence, if $R_0>1$, there exist a unique positive solution which is $E^*$. $\square$

Next, we study the local stability of the disease-free equilibrium $E_f$ and the endemic equilibrium $E^*$. The Jacobian matrix of system (4.3) at any equilibrium $\bar{E} = (\bar{S}, \bar{I})$ is given by

5.1 $$\begin{aligned} J_{\bar{E}} =\left( \begin{array}{ccc} -\mu -u-\beta \bar{I} &{} -\beta \bar{S} \\ \beta \bar{I} &{} \beta \bar{S}-(\mu +r) \end{array} \right) . \end{aligned}$$

We recall that a sufficient condition for the local stability of $\bar{E}$ is

5.2 $$\begin{aligned} |arg(\xi _i)| > \dfrac{\alpha \pi }{2}, \quad i=1, 2, \end{aligned}$$

where $\xi _i$ are the eigenvalues of $J_{\bar{E}}$ (see^27^). First, we establish the local stability of $E_f$.

### Theorem 5.2

If $R_0 < 1$, then the disease-free equilibrium $E_f$ is locally asymptotically stable.

### Proof

At $$E_f$$, (5.1) becomes

$$\begin{aligned} J_{E_f}= \left( \begin{array}{ccc} -\mu -u &{} -\beta S_f \\ 0 &{} \beta S_f-(\mu +r) \end{array} \right) . \end{aligned}$$

Hence, the eigenvalues of $J_{E_f}$ are $\xi _{1} = -\mu -u$, $\xi _{2} = (\mu +r) (R_{0} - 1)$. Clearly, $\xi _2$ satisfies condition (5.2) if $R_0 < 1$, since $\xi _1$ is negative, proving the desired result. $\square$

We now establish the local stability of $E^*$.

### Theorem 5.3

If $R_0 > 1$, then the endemic equilibrium $E^*$ is locally asymptotically stable.

### Proof

At equilibrium $E^*$, the characteristic equation for the corresponding linearised system of model (4.3) is $\xi ^2 +a_1 \xi +a_2 =0$, where

$$\begin{aligned} a_1 = (\mu +u)R_0, \end{aligned}$$

and

$$\begin{aligned} a_2 = (\mu +r)(\mu +u)(R_0-1). \end{aligned}$$

If $R_0>1$ then $a_1>0$ and $a_2>0$. From^28^, we have the desired result. $\square$

## Global stability

Our goal now is to study the global behavior for $E_f$ and $E^*$ using Lyapunov’s functions. Firstly we give the definition of Lyapunov function. Consider the following fractional differential equation:

6.1 $$\begin{aligned} {\mathcal {D}}_t^\alpha u (t) = f (u (t)), \end{aligned}$$

with the initial condition:

$$\begin{aligned} u (0) = u_0, \end{aligned}$$

where $$ {\mathcal {D}}_t^\alpha $$ is the fractional derivative in Caputo sense of order $$ \alpha \in (0,1] $$, the state variable is a positive vector of *m* elements $$ u_1 ,\ldots , u_m $$, and $$ f: \mathbb {R}^m \longrightarrow \mathbb {R}^m $$ is a function of class $$ C^1$$.

### Definition 6.1

(^29^) Let $u^*$ be an equilibrium point of system (6.1) such that $\Theta (u^*)$ a neighborhood of $u^*$. Let V be a differentiable function defined on $\Theta (u^*)$ and with real value. We say that V is a Lyapunov function in $u ^ *$, if it satisfies the following two properties: $V (u^*) = 0$ and $V (u)> 0$ in $\Theta (u^*)$ for all $u \ne u^*$.

### Theorem 6.2

(LaSalle principle^30^). Let $u^*$ be an equilibrium point of the system (6.1) and let *V* be a positive function of class $C^1$ defined in the neighborhood $\Theta (u^*)$ of $u^*$. Then $u^*$ is asymptotically stable if:

1. $$ {\mathcal {D}}_t ^ \alpha V (u) \le 0 $$    for all $$ u \in \Theta (u^*) $$.
2. The set $\{u \in \Theta (u^*);\, \, {\mathcal {D}}_t^\alpha V = 0 \}$ contains no other trajectory other than $u^*$.

Moreover, if $V(u) \rightarrow \infty$, when $\Vert u \Vert \rightarrow \infty$, then $u^*$ is globally asymptotically stable.

Secondly, to prove the global stability of *DFE*, we need to use the following auxiliary lemma for the purpose of the application of Lyapunov function in the case of the fractional order systems:

### Lemma 6.3

(^31^). We put $y(t)\in \mathbb {R^*_+}$ be a continuous and derivable function. For all $\alpha \in (0,1)$ and for $t\ge t_0$

$$\begin{aligned} ^{C}_{t_0}D{_t^{\alpha }}\bigg [y^*\Psi \bigg (\frac{y(t)}{y^*}\bigg )\bigg ]\le \bigg (1-\frac{y^*}{y(t)}\bigg )\,^{C}_{t_0}D{_t^{\alpha }}y(t),\, y^*\in \mathbb {R^*_+}, \end{aligned}$$

where $\Psi$ is a positive function defined by $\Psi (y) = - \ln (y)+y-1$, $y > 0$.

### Theorem 6.4

$E_f$ is globally asymptotically stable for $R_0 \le 1$.

### Proof

Introducing the Lyapunov function:

$$\begin{aligned} V(t)=\int _{\Omega }\bigg (S_f\Psi \bigg (\frac{S{(t,x)}}{S_f}\bigg )+I{(t,x)}\bigg )dx. \end{aligned}$$

Calculating the fractional derivative of *V* in Caputo’s sense, we have

$$\begin{aligned} ^{C}_{0}D{_t^{\alpha }}V(t)&\le \int _{\Omega }\bigg (\big (1-\frac{S_f}{S{(t,x)}}\big )\,^{C}_{0}D{_t^{\alpha }}S{(t,x)}+\,^{C}_{0}D{_t^{\alpha }}I{(t,x)}\bigg )dx\\ \quad&\le \int _{\Omega }\bigg (\big (1-\frac{S_f}{S{(t,x)}}\big )\big (\Lambda -\beta S{(t,x)}I{(t,x)}-(\mu +u)S{(t,x)}\big )\\&\quad +\beta S{(t,x)}I{(t,x)}-(\mu +r)I{(t,x)}\bigg )dx\\&\quad +\int _{\Omega }\bigg (\lambda _1 \Delta S{(t,x)}-\lambda _1\frac{S_f}{S{(t,x)}}\Delta S{(t,x)}+\lambda _2\Delta I{(t,x)}\bigg )dx. \end{aligned}$$

Since $$\Lambda =(\mu +u)S_f$$, then

$$\begin{aligned} ^{C}_{0}D{_t^{\alpha }}V(t)&\le \int _{\Omega }\bigg (\big (1-\frac{S_f}{S{(t,x)}}\big )\,\big ((\mu +u)S_f-(\mu +u)S{(t,x)}\big )\\&\quad +\frac{\beta \Lambda }{\mu +u}I{(t,x)}-(\mu +r)I{(t,x)}\bigg )dx\\&\quad +\int _{\Omega }\bigg (\lambda _1 \Delta S{(t,x)}-\lambda _1\frac{S_f}{S{(t,x)}}\Delta S{(t,x)}+\lambda _2\Delta I{(t,x)}\bigg )dx. \end{aligned}$$

Applying Green’s formula, we get

$$\begin{aligned} ^{C}_{0}D{_t^{\alpha }}V(t)&\le -(\mu +u)\int _{\Omega }\frac{(S{(t,x)}-S_f)^2}{S{(t,x)}}dx+(\mu +r)\int _{\Omega }(R_0-1)I{(t,x)}dx\\&\quad -\lambda _1 S_f \int _{\Omega }\frac{|\nabla S{(t,x)}|^2}{S^2{(t,x)}}dx. \end{aligned}$$

For $R_0\le 1$, we deduce that $^{C}_{0}D{_t^{\alpha }}V(t)\le 0$. In addition $^{C}_{0}D{_t^{\alpha }}V(t)=0$ is equivalent to $S=S_f$ and $(R_0-1)I=0$. Then the following two cases arise:

- If $$R_0<1$$, then $$I=0$$.
- If $R_0=1$, using the first eq. of (4.3) together with $S=S_f$ , we get
  $$\begin{aligned} \Lambda -(\mu +u)S_f-\beta S_f I{(t,x)}= 0\,, \end{aligned}$$
  then $\beta S_f I{(t,x)}= 0$. Thus, we obtain $I=0$.

Hence, the largest invariant set of $\left\{ (S,I)\in \mathbb {R}_+^2 :\, ^{C}_{0}D{_t^{\alpha }} V(t) = 0\right\}$ is the singleton $\{E_f\}$. Using LaSalle’s invariance principle^30^, we conclude that ${E_f}$ is globally asymptotically stable. $\square$

Similarly, we shall show global stability of $E^*$ which is resumed in the following theorem

### Theorem 6.5

$E^*$ is globally asymptotically stable whenever exists.

### Proof

We consider the Lyapunov function:

$$\begin{aligned} V(t)=\int _{\Omega }\bigg (S^*\Psi \bigg (\frac{S{(t,x)}}{S^*}\bigg )+ I^*\Psi \bigg (\frac{I{(t,x)}}{I^*}\bigg )\bigg )dx. \end{aligned}$$

We have

$$\begin{aligned} ^{C}_{0}D{_t^{\alpha }}V(t)&\le \bigg (1-\frac{S^*}{S{(t,x)}}\bigg )\, ^{C}_{0}D{_t^{\alpha }}S{(t,x)}+\bigg (1-\frac{I^*}{I{(t,x)}}\bigg )\, ^{C}_{0}D{_t^{\alpha }}I{(t,x)}\\ \quad&\le \int _{\Omega }\bigg (\big (1-\frac{S^*}{S{(t,x)}}\big )\big (\Lambda -\beta S{(t,x)}I{(t,x)}-(\mu +u)S{(t,x)}\big )\\&\quad +\big (1-\frac{I^*}{I{(t,x)}}\big )\big (\beta S{(t,x)}I{(t,x)}-(\mu +r)I{(t,x)}\big )\bigg )dx +\int _{\Omega }\bigg (\lambda _1 \Delta S{(t,x)}\\&\quad -\lambda _1\frac{S^*}{S{(t,x)}}\Delta S{(t,x)}+\lambda _2\Delta I{(t,x)}-\lambda _2\frac{I^*}{I{(t,x)}}\Delta I{(t,x)}\bigg )dx. \end{aligned}$$

Note that $\mu +r=\beta S^*$, $\Lambda =(\mu +u)S^*+(\mu +r)I^*$. Applying then Green’s formula, we obtain

$$\begin{aligned} ^{C}_{0}D{_t^{\alpha }}V(t)&\le -(\mu +u)\int _{\Omega }\frac{(S{(t,x)}-S^*)^2}{S{(t,x)}}dx+\int _{\Omega }\bigg (2(\mu +r)I^*-(\mu +r)I^*\frac{S^*}{S{(t,x)}}\\ \quad&-(\mu +r)I^*\frac{S{(t,x)}}{S^*}\bigg )dx -\lambda _1 S^* \int _{\Omega }\frac{|\nabla S{(t,x)}|^2}{S^2{(t,x)}}dx-\lambda _2 I^* \int _{\Omega }\frac{|\nabla I{(t,x)}|^2}{I^2{(t,x)}}dx\\ \quad&\le -(\mu +u)\int _{\Omega }\frac{(S{(t,x)}-S^*)^2}{S{(t,x)}}dx+\int _{\Omega }(\mu +r)I^*\bigg (2-\frac{S^*}{S{(t,x)}}\\&\quad -\frac{S{(t,x)}}{S^*}\bigg )dx-\lambda _1 S^* \int _{\Omega }\frac{|\nabla S{(t,x)}|^2}{S^2{(t,x)}}dx-\lambda _2 I^* \int _{\Omega }\frac{|\nabla I{(t,x)}|^2}{I^2{(t,x)}}dx\\ \quad&\le -(\mu +u)\int _{\Omega }\frac{(S{(t,x)}-S^*)^2}{S{(t,x)}}dx-(\mu +r)I^*\int _{\Omega }\Psi \bigg (\frac{S^*}{S{(t,x)}}\bigg )dx\\&\quad -(\mu +r)I^*\int _{\Omega }\Psi \bigg (\frac{S{(t,x)}}{S^*}\bigg )dx-\lambda _1 S^* \int _{\Omega }\frac{|\nabla S{(t,x)}|^2}{S^2{(t,x)}}dx\\&\quad -\lambda _2 I^* \int _{\Omega }\frac{|\nabla I{(t,x)}|^2}{I^2{(t,x)}}dx. \end{aligned}$$

Since $\Psi (y) \ge 0$, then $D^{\alpha }V(t) \le 0$ for $R_0 > 0$. Furthermore, the largest invariant set that verifies $\{(S,I)\in \mathbb {R}_+^2 : D^\alpha V(t) = 0\}$ is $\{E^*\}$. Using LaSalle’s we achieve the desired result. $\square$

## Graphical representation

In this section, we present some graphical illustrations confirming our theoretical findings. The system (3.2)–(3.4) is numerically integrated by using the forward finite difference approximations to discretize the time-fractional derivative^32^ and the centered finite difference schemes to approach the Laplacian’s operator in one-dimensional space, then we can take $\Omega =[0, L]$. This method gives an accurate of order $2- \alpha$ in time and order 2 in space^32^.

Let $\delta =\frac{T}{N}$ and $\Delta x=\frac{L}{n}$ be the length of each time step and the space step respectively, for some large N and n, $t_l=l\delta$ for $l=0,\ldots ,N$ and $x_i=i\Delta x$ for $i=0,\ldots ,n.$ We have

$$\begin{aligned} ^{C}_{0}D{_t^{\alpha }}S{(t_l,x_i)}\approx \frac{1}{\Gamma (2-\alpha )}\sum _{j=0}^{l}\frac{(j+1)^{1-\alpha }-j^{1-\alpha }}{\delta ^{\alpha }}\bigg (S(t_{l+1-j},x_i)-S(t_{l-j},x_i)\bigg ), \end{aligned}$$

and

$$\begin{aligned} \Delta S{(t_l,x_i)}\approx \frac{S(t_l,x_{i+1})-2S(t_l,x_{i})+S(t_l,x_{i-1})}{\Delta x}. \end{aligned}$$

Then, we obtain the following scheme:

7.1 $$\begin{aligned} \begin{aligned} S^{l+1}_{i}&=S^{l}_{i}-\sum _{j=1}^{l}\big ((j+1)^{1-\alpha }-j^{1-\alpha }\big )\big (S^{l+1-j}_{i}-S^{l-j}_{i}\big )+\frac{\lambda _1\times \Gamma (2-\alpha )\times \delta ^{\alpha }}{\Delta X^2}\big (S^{l}_{i+1}-2S^{l}_{i}+S^{l}_{i-1}\big )\\&\quad +\Gamma (2-\alpha )\times \delta ^{\alpha }\times f_{1i}^l\, ,\\ I^{l+1}_{i}&=I^{l}_{i}-\sum _{j=1}^{l}\big ((j+1)^{1-\alpha }-j^{1-\alpha }\big )\big (I^{l+1-j}_{i}-I^{l-j}_{i}\big )+\frac{\lambda _1\times \Gamma (2-\alpha )\times \delta ^{\alpha }}{\Delta X^2}\big (I^{l}_{i+1}-2I^{l}_{i}+I^{l}_{i-1}\big )\\&\quad +\Gamma (2-\alpha )\times \delta ^{\alpha }\times f_{2i}^l\, ,\\ R^{l+1}_{i}&=R^{l}_{i}-\sum _{j=1}^{l}\big ((j+1)^{1-\alpha }-j^{1-\alpha }\big )\big (R^{l+1-j}_{i}-R^{l-j}_{i}\big )+\frac{\lambda _1\times \Gamma (2-\alpha )\times \delta ^{\alpha }}{\Delta X^2}\big (R^{l}_{i+1}-2R^{l}_{i}+R^{l}_{i-1}\big )\\&\quad +\Gamma (2-\alpha )\times \delta ^{\alpha }\times f_{3i}^l\, , \end{aligned} \end{aligned}$$

with

7.2 $$\begin{aligned} \begin{aligned} f_{1i}^l&=\Lambda -\beta S^{l}_{i} I^{l}_{i}-\mu S^{l}_{i}-uS^{l}_{i}\, ,\\ f_{2i}^l&=\beta S^{l}_{i} I^{l}_{i}-(\mu +r)I^{l}_{i}\, ,\\ f_{3i}^l&=r I^{l}_{i}- \mu R^{l}_{i} + uS^{l}_{i}\, . \end{aligned} \end{aligned}$$

Note that unlike the usual derivative, the fractional derivatives are not local operators, i.e. for example to calculate $S^{l}_{i}$ the number of susceptible at time *l*, we need all of its information up to the initial instant, and that comes from the summation term $\sum _{j=1}^{l}\big ((j+1)^{1-\alpha }-j^{1-\alpha }\big )\big (S^{l+1-j}_{i}-S^{l-j}_{i}\big )$ which represents the memory effect. We also notice when $\alpha =1$ we obtain the discretization of classical model without memory.

**Figure 1:**
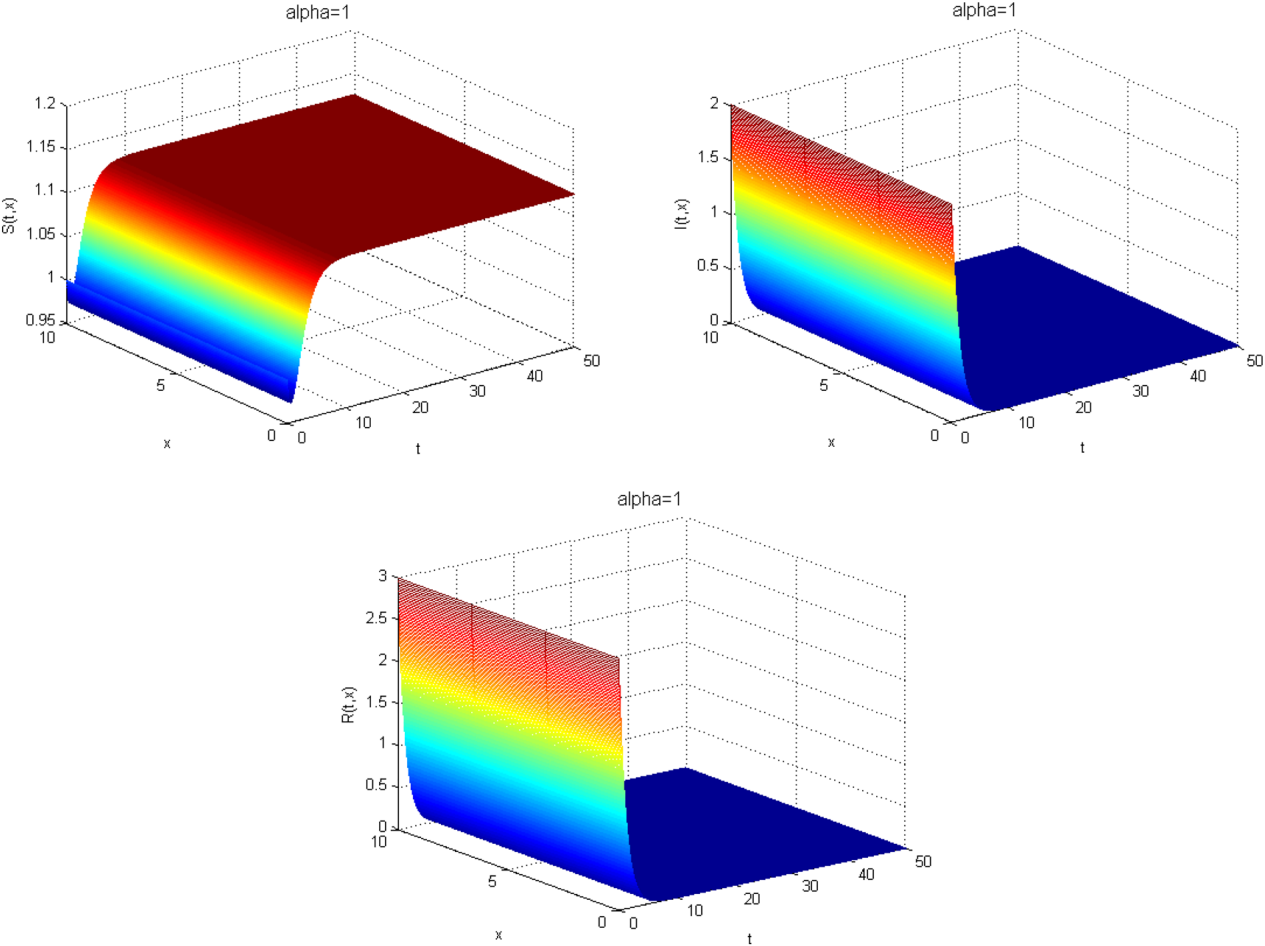
Dynamics of the system (3.2) for $\alpha =1$.

**Figure 2:**
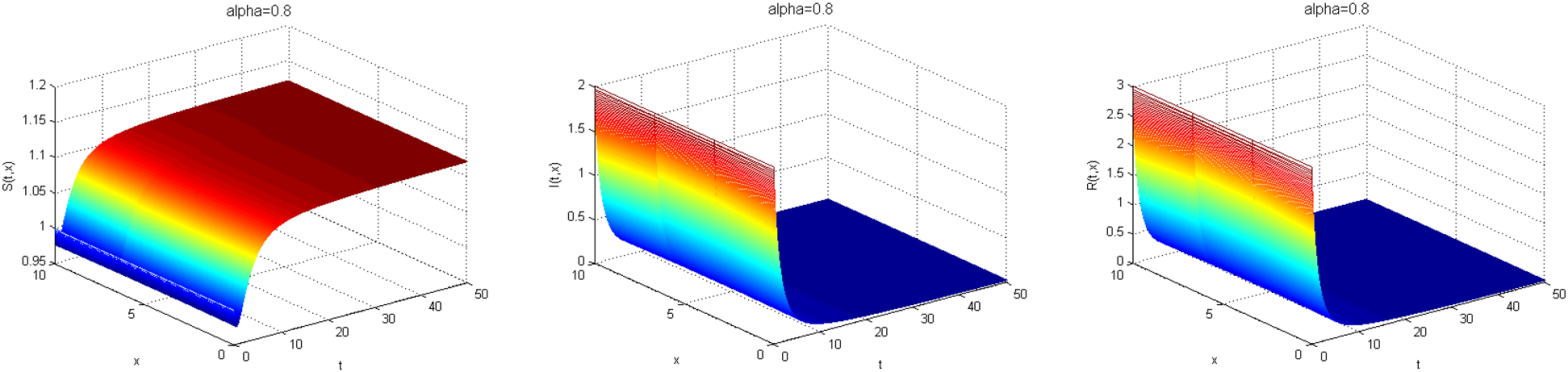
Dynamics of the system (3.2) for $\alpha =0.8$.

**Figure 3:**
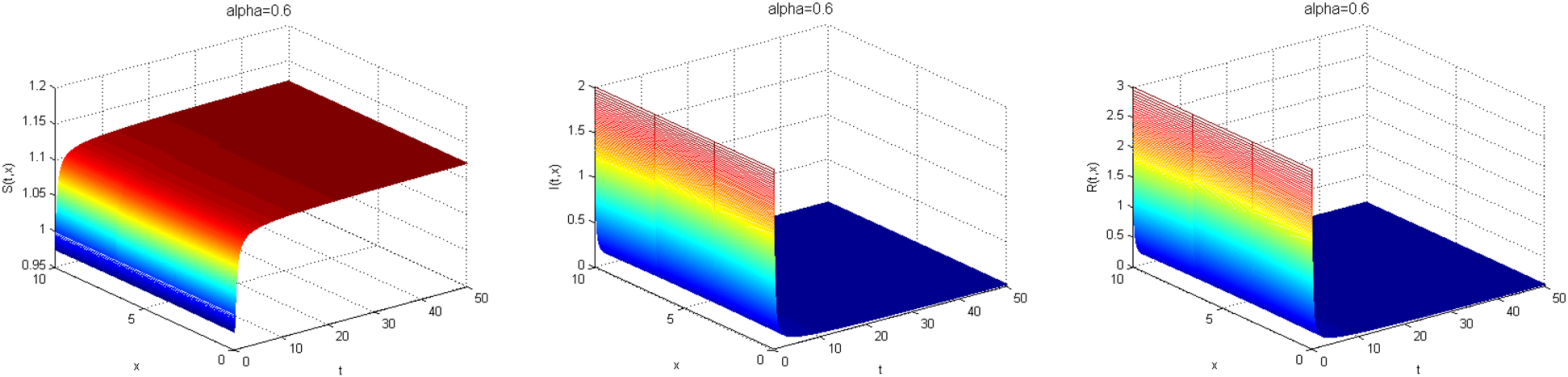
Dynamics of the system (3.2) for $\alpha =0.6$.

**Figure 4:**
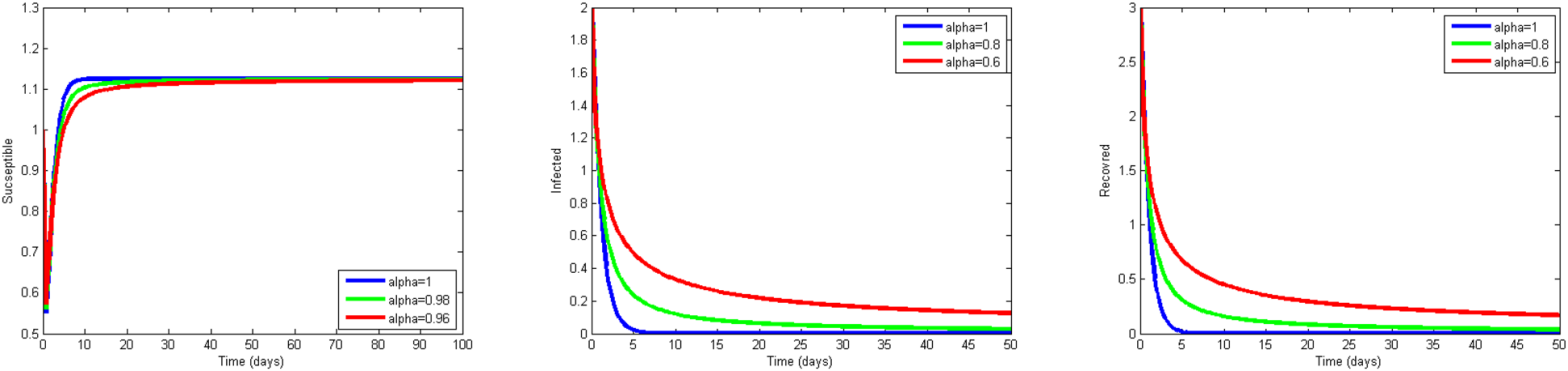
Dynamics of the system (3.2) for x fixed and for $\alpha =0.6, 0.8, 1$ in the case where $R_0 = 0.1372<1$.

Next, we study the case without vaccination. Let $$ L = 10$$ and $$ T = 50$$. We simulate system (3.2)–(3.4) with the following set of parameters: $$\mu = 0.8$$, $$\Lambda = 0.9$$, $$\beta = 0.1$$, $$r = 0.02$$, $$u=0.0$$, $$\lambda _1=\lambda _2=\lambda _3=0.2$$, and the initial conditions $$S(0,x) = 1.0$$, $$I(0,x) = 2.0$$ and $$R(0,x) = 3.0$$. As a result we approximate the solutions of (3.2)–(3.4) for $$\alpha =1$$, $$\alpha =0.8$$ and $$\alpha =0.6$$ that displayed respectively in Figs. 1, 2 and 3. We also calculate $$R_0 = 0.1372$$. Hence, system (3.2)–(3.4) has a unique equilibrium $$E_f = (1.12, 0, 0)$$. Using Theorem 6.4, $$E_f$$ is globally stable. In Fig. 4, we have fixed the space variable *x* to show the effect of the order $$ \alpha $$ along the dynamics of the solution. We notice that all the solutions are globally asymptotically stable for different values of $$ \alpha $$ not just for $$ \alpha = 1 $$. We also notice that the solution for $$ \alpha = 1 $$ quickly converges to the equilibrium point $$E_f$$. Since fractional derivatives describe reality well, we can say that the epidemic takes a longer duration to be stable. This is very important in terms of economics and the study of control strategies.

**Figure 5:**
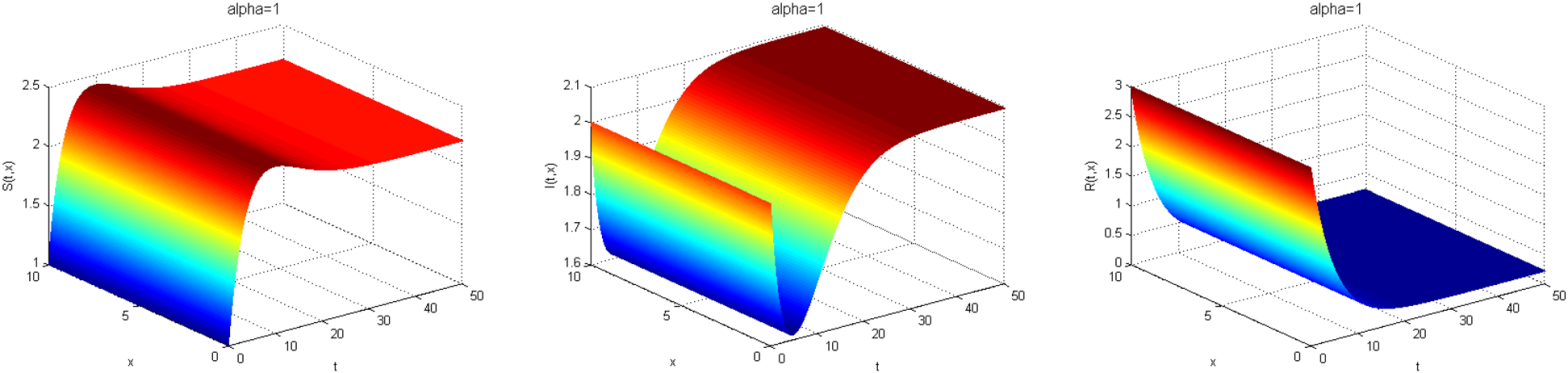
Dynamics of the system (3.2) for $\alpha =1$.

**Figure 6:**
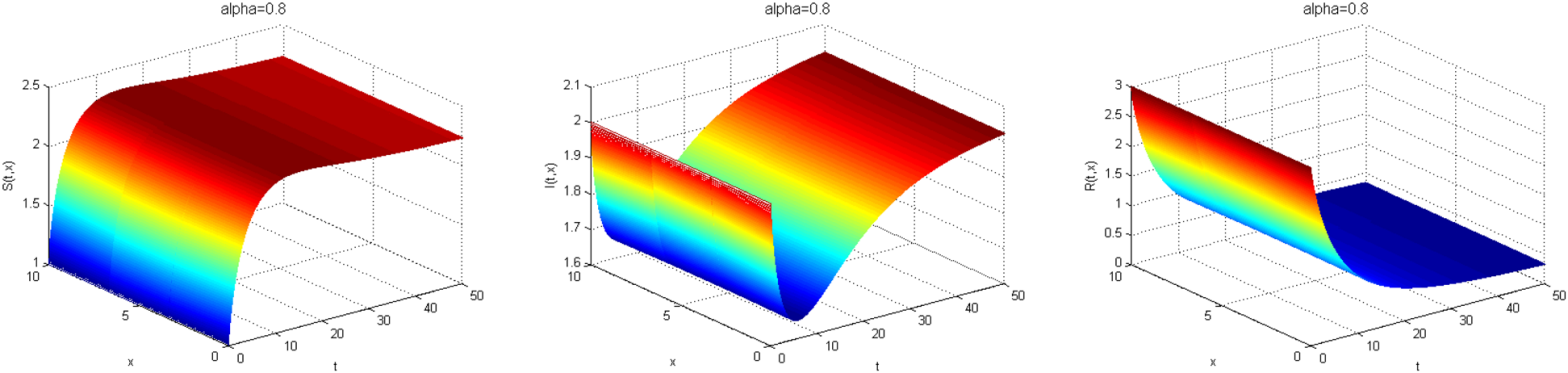
Dynamics of the system (3.2) for $\alpha =0.8$.

**Figure 7:**
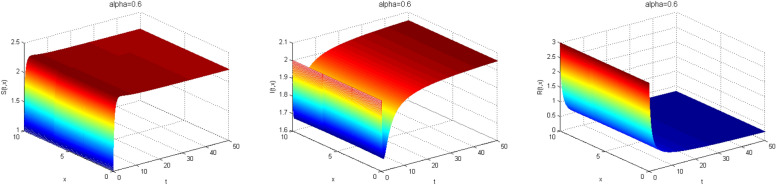
Dynamics of the system (3.2) for $\alpha =0.6$.

**Figure 8:**
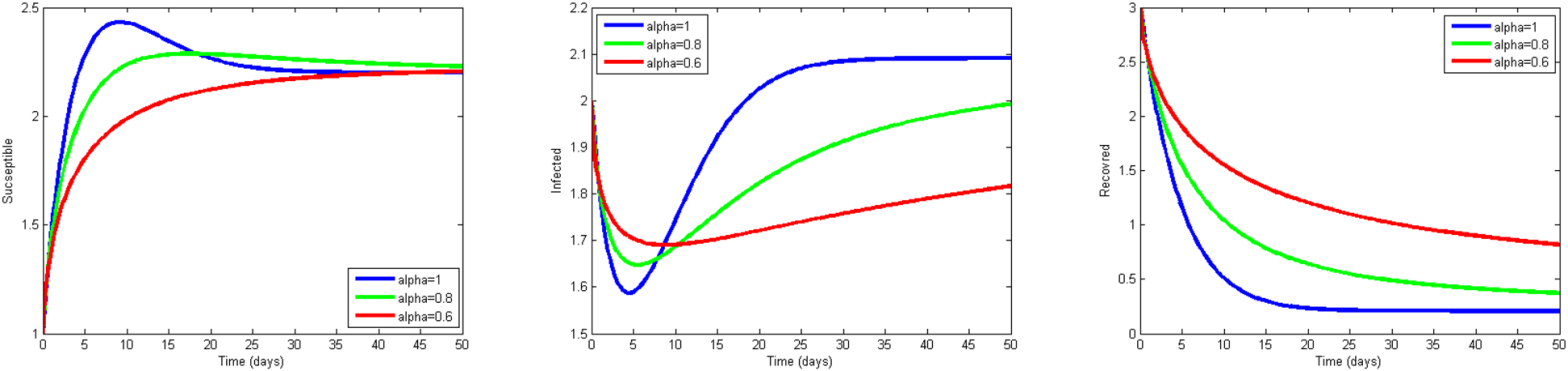
Dynamics of the system (3.2) for x fixed and for $\alpha =0.6, 0.8, 1$ in the case where $R_0 = 2.0455>1$.

Now, we consider $\mu = 0.2$ and letting the same previous set of parameter. Then, $R_0 = 2.0455$. From Theorem 6.5, $E^*$ is globally asymptotically stable. Figures 5, 6 and 7 illustrate this result for different values of $\alpha$, which means biologically that the infection persists but it is under control. For easy comparison see Fig. 8.

**Figure 9:**
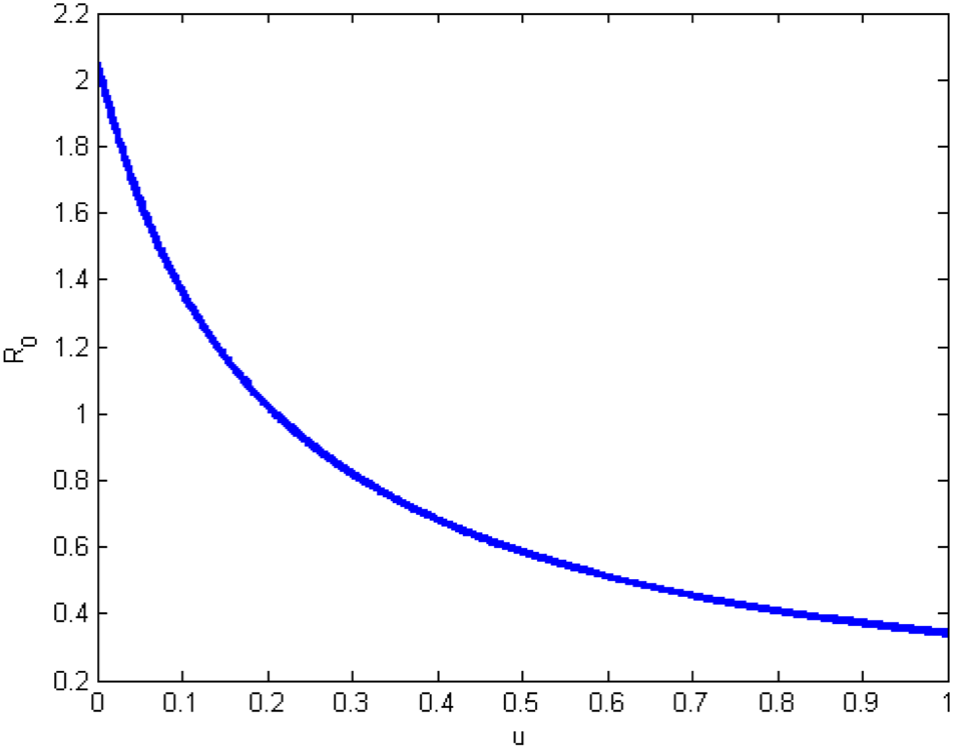
Variation of $R_0$ according to the vaccine effect.

**Figure 10:**
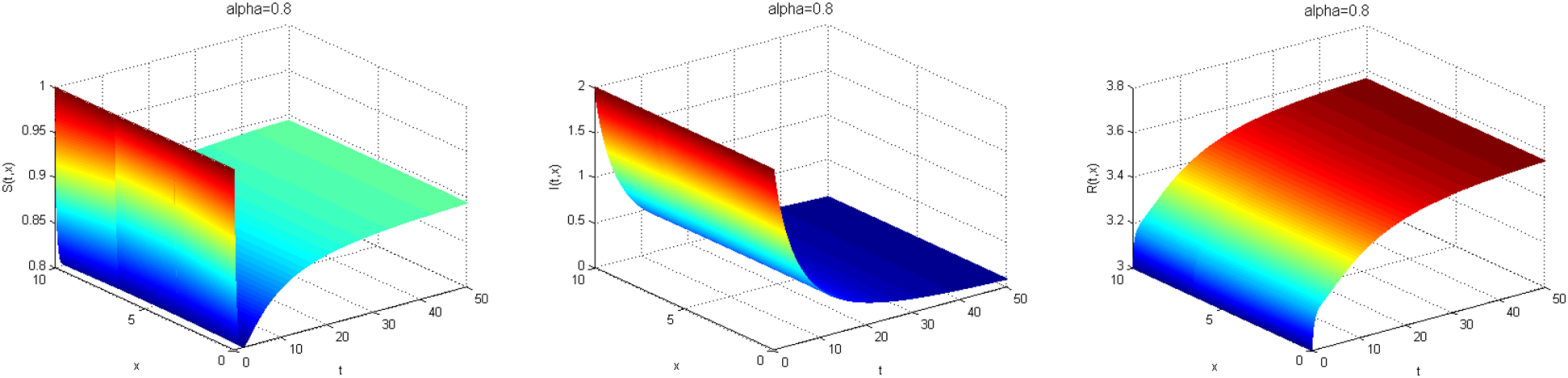
Behavior of the solution for $\alpha = 0.8$ under the effect of vaccine ($u=0.8$).

Finally, let’s study the effect of the vaccine on the basic reproduction rate $R_0$. We are only interested in the endemic case for $\alpha =0.8$. We note that the basic reproduction rate decreases when the vaccine rate increases (for example $u = 0.8$ we have $R_0= 0.4091$ (see Fig. 9)). Consequently the number of infected individuals is also decreasing. Besides, the number of removed individuals increases at the expense of susceptible people (see Fig. 10). It reflects the importance of the vaccine to eradicate the disease.

## Conclusion

We dealt in this paper with the qualitative behavior of the solutions of a reaction–diffusion system under the influence of the fractional derivative $\alpha$. Firstly, we investigated the global behavior of more real extension’s of a basic *SIR* model with memory effects measured by Caputo’s fractional derivative in time of order $0 <\alpha \le 1$, such that when $\alpha = 1$ we obtain the classical model (without memory). Secondly, we have constructed Lyapunov functions to show the global stability of the equilibrium points in a more general framework where the proposed system takes into account the spatial behavior of populations and memory effect. Taking advantage of the Lyapunov function method we have shown that $R_0$ plays an important role in determining the global dynamics of the proposed model. We have established the global stability of the two equilibria: $E_f$ and $E^*$ for different values of $\alpha$. From epidemiological point of view, this means that the infection will eradicated or persisted while respecting certain restrictions on the parameters. According to our theoretical analysis, we obtained the stability of the equilibria not only for the integer derivative ($\alpha = 1$) but also for all $0 <\alpha \le 1$, which confirms the generality of our system. In addition, fractional derivatives have provided other means of predicting the progression of the disease and, in some cases, affecting the time required to reach stable states. Our future work is to control the vaccination term *u* to get a better optimal strategy with other fractional derivatives having a non singular kernel.

## Methods

As an application of the fractional derivatives a diffusive *SIR* epidemic model is described. The fixed point theory is adopted for the results related existence and uniqueness of the solution and Lyapunov function theory is utilized for the stability analysis of proposed model. Numerical results are done for the verification of obtained results and it is surety that it will help the researcher in future related fractional order models.

## Data Availability

The database used and analyzed during the current study are available from the corresponding author on reasonable request.
